# Di-μ_2_-methano­lato-bis­(μ-4-methyl-5-sulfanyl­idene-4,5-dihydro-1*H*-1,2,4-triazolido-κ^2^ *N* ^1^:*N* ^2^)di-μ_3_-oxido-tetra­kis­[dimethyl­tin(IV)]

**DOI:** 10.1107/S1600536811001905

**Published:** 2011-01-22

**Authors:** Ezzatollah Najafi, Mostafa M. Amini, Seik Weng Ng

**Affiliations:** a Department of Chemistry, General Campus, Shahid Beheshti University, Tehran 1983963113, Iran; b Department of Chemistry, University of Malaya, 50603 Kuala Lumpur, Malaysia

## Abstract

The title distannoxane, [Sn_4_(CH_3_)_8_(C_3_H_4_N_3_S)_2_(CH_3_O)_2_O_2_], lies about a center of inversion; the tetra­nuclear mol­ecule features a three-rung-staircase Sn_4_O_4_ core in which the two crystallographically independent Sn^IV^ atoms are bridged by the triazolide group. The negatively charged N atom of the triazolide group binds to the terminal Sn atom at a shorter distance [Sn—N = 2.239 (2) Å] compared with the neutral N atom that binds to the central Sn atom [Sn← N = 2.757 (3) Å]. The oxide O atom is three-coordinate whereas the methano­late O atom is two-coordinate. The terminal Sn atom is five-coordinate in a *cis*-C_3_SnNO trigonal–bipyramidal environment, whereas the central Sn atom is six-coordinate in a C_2_SnNO_3_ skew-trapezoidal–bipyramidal geometry.

## Related literature

For related distannoxanes, see: Ma *et al.* (2007 ▶); Yu *et al.* (2006 ▶).
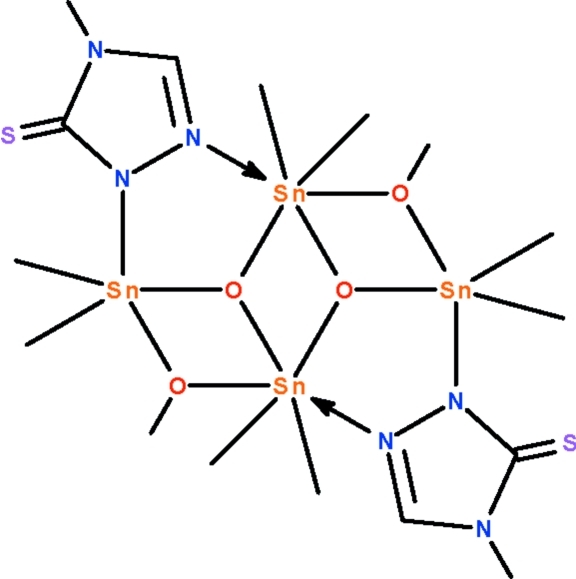


## Experimental

### 

#### Crystal data

[Sn_4_(CH_3_)_8_(C_3_H_4_N_3_S)_2_(CH_3_O)_2_O_2_]
*M* *_r_* = 917.40
Triclinic, 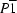

*a* = 7.3693 (6) Å
*b* = 9.3457 (8) Å
*c* = 11.9930 (9) Å
α = 71.681 (7)°
β = 76.780 (6)°
γ = 77.118 (7)°
*V* = 753.07 (11) Å^3^
*Z* = 1
Mo *K*α radiation
μ = 3.45 mm^−1^
*T* = 100 K
0.30 × 0.25 × 0.20 mm

#### Data collection

Agilent Technologies SuperNova Dual diffractometer with an Atlas detector
Absorption correction: multi-scan (*CrysAlis PRO*; Agilent Technologies, 2010 ▶) *T*_min_ = 0.425, *T*_max_ = 0.546
5805 measured reflections
3328 independent reflections
2919 reflections with *I* > 2σ(*I*)
*R*_int_ = 0.029

#### Refinement

*R*[*F* ^2^ > 2σ(*F* ^2^)] = 0.024
*wR*(*F* ^2^) = 0.047
*S* = 0.98
3328 reflections
152 parameters
H-atom parameters constrained
Δρ_max_ = 0.83 e Å^−3^
Δρ_min_ = −0.77 e Å^−3^

### 

Data collection: *CrysAlis PRO* (Agilent Technologies, 2010 ▶); cell refinement: *CrysAlis PRO*; data reduction: *CrysAlis PRO*; program(s) used to solve structure: *SHELXS97* (Sheldrick, 2008 ▶); program(s) used to refine structure: *SHELXL97* (Sheldrick, 2008 ▶); molecular graphics: *X-SEED* (Barbour, 2001 ▶); software used to prepare material for publication: *publCIF* (Westrip, 2010 ▶).

## Supplementary Material

Crystal structure: contains datablocks global, I. DOI: 10.1107/S1600536811001905/xu5141sup1.cif

Structure factors: contains datablocks I. DOI: 10.1107/S1600536811001905/xu5141Isup2.hkl

Additional supplementary materials:  crystallographic information; 3D view; checkCIF report

## Figures and Tables

**Fig. 1.**
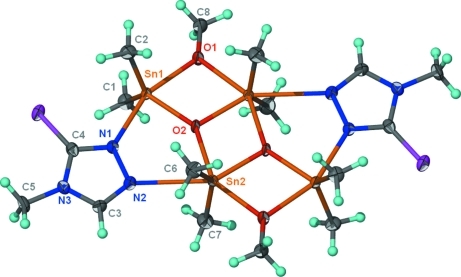
Thermal ellipsoid plot (Barbour, 2001) of Sn4O2(CH3)8(CH3O)2(C3H4N3S)2 at the 70% probability level; hydrogen atoms are drawn as spheres of arbitrary radius.

## supplementary crystallographic information

### Comment

The title compound (Scheme I, Fig. 1), a distannoxane, was the unexpected product from an attempt at synthesizing a dimethyltin 4,-methyl-4*H*-1,2,4-triazol-3-thiolate that possesses a tin-sulfur linkage. In the reaction of diorganotin oxides with organic acids (particularly carboxylic acid), tetranuclear distannoxanes are sometimes formed; these compounds have four organic groups. In the present reaction, two of the four organic groups are replaced by methoxide groups.

Tetranuclear Sn_4_O_2_(CH_3_)_8_(CH_3_O)_2_(C_3_H_4_N_3_S)_2_ distannoxane lies about a center-of-inversion; the molecule features a three-rung-staircase Sn_4_O_4_ core in which two Sn atoms are bridged by the C_3_H_4_N_3_S triazolide group. The negatively-charged N atom of the group binds to the terminal Sn atom at a shorter distance [Sn–N 2.239 (2) Å] compared with the neutral N atom that binds to the central Sn atom [Sn←N 2.757 (2) Å]. The oxo O atom is three-coordinate whereas the methanolate O atom is two-coordinate. The terminal Sn atom is five-coordinate in a *cis*-C_3_SnNO trigonal bipyramid whereas the central Sn atom is six-coordinate in a C_2_SnNO_3_ skew-trazepoidal bipyramidal geometry.

The formation of similar distannoxanes that feature bridging triazolides are limited to the 4-(benzylideneamino)-3-methyl-5-thioxo-1,2,4-triazolide, 4-(2-furylmethylene)amino-3-methyl-5-thioxo-1,2,4-triazolide, 4-(2-thienylmethylene)amino-3-methyl-5-thioxo-1,2,4-triazolide and 5-(2-thienylmethylene)amino-2-thioxo-1,3,4-thiadiazolates only (Ma *et al.*, 2007; Yu *et al.*, 2006).

### Experimental

Dimethyltin diisothiocyanate (1 mmol), 4-methyl-4*H*-1,2,4-triazole-3-thiol (1 mmol) and 1,10-phenanthroline (1 mmol) were loaded into a convection tube; several drops of triethylamine were added. The tube was filled with dry methanol and kept at 333 K. Colorless crystals were collected from the side arm after several days.

### Refinement

Carbon-bound H-atoms were placed in calculated positions [C—H 0.95 to 0.98 Å, *U*_iso_(H) 1.2 to 1.5*U*_eq_(C)] and were included in the refinement in the riding model approximation.

### Crystal data

**Table:**

[Sn_4_(CH_3_)_8_(C_3_H_4_N_3_S)_2_(CH_3_O)_2_O_2_]	*Z* = 1
*M**_r_* = 917.40	*F*(000) = 440
Triclinic, *P*1	*D*_x_ = 2.023 Mg m^−^^3^
Hall symbol: -P 1	Mo *K*α radiation, λ = 0.71073 Å
*a* = 7.3693 (6) Å	Cell parameters from 3945 reflections
*b* = 9.3457 (8) Å	θ = 2.3–29.2°
*c* = 11.9930 (9) Å	µ = 3.45 mm^−^^1^
α = 71.681 (7)°	*T* = 100 K
β = 76.780 (6)°	Block, colorless
γ = 77.118 (7)°	0.30 × 0.25 × 0.20 mm
*V* = 753.07 (11) Å^3^	

### Data collection

**Table:**

Agilent SuperNova Dual diffractometer with an Atlas detector	3328 independent reflections
Radiation source: SuperNova (Mo) X-ray Source	2919 reflections with *I* > 2σ(*I*)
Mirror	*R*_int_ = 0.029
Detector resolution: 10.4041 pixels mm^-1^	θ_max_ = 27.5°, θ_min_ = 2.3°
ω scans	*h* = −7→9
Absorption correction: multi-scan (*CrysAlis PRO*; Agilent, 2010)	*k* = −12→11
*T*_min_ = 0.425, *T*_max_ = 0.546	*l* = −14→15
5805 measured reflections	

### Refinement

**Table:**

Refinement on *F*^2^	Secondary atom site location: difference Fourier map
Least-squares matrix: full	Hydrogen site location: inferred from neighbouring sites
*R*[*F*^2^ > 2σ(*F*^2^)] = 0.024	H-atom parameters constrained
*wR*(*F*^2^) = 0.047	*w* = 1/[σ^2^(*F*_o_^2^) + (0.0144*P*)^2^] where *P* = (*F*_o_^2^ + 2*F*_c_^2^)/3
*S* = 0.98	(Δ/σ)_max_ = 0.001
3328 reflections	Δρ_max_ = 0.83 e Å^−^^3^
152 parameters	Δρ_min_ = −0.77 e Å^−^^3^
0 restraints	Extinction correction: *SHELXL97* (Sheldrick, 2008), Fc^*^=kFc[1+0.001xFc^2^λ^3^/sin(2θ)]^-1/4^
Primary atom site location: structure-invariant direct methods	Extinction coefficient: 0.0086 (3)

### Fractional atomic coordinates and isotropic or equivalent isotropic displacement parameters (Å^2^)

**Table:**

	*x*	*y*	*z*	*U*_iso_*/*U*_eq_	
Sn1	0.23531 (3)	0.50079 (2)	0.258264 (16)	0.01270 (7)	
Sn2	0.57195 (3)	0.31342 (2)	0.021831 (16)	0.01318 (7)	
S1	0.14046 (14)	0.21699 (10)	0.54808 (7)	0.0233 (2)	
O1	0.2321 (3)	0.7285 (2)	0.13758 (16)	0.0188 (5)	
O2	0.3998 (3)	0.4777 (2)	0.10279 (16)	0.0154 (5)	
N1	0.3105 (4)	0.2459 (3)	0.3171 (2)	0.0156 (6)	
N2	0.4081 (4)	0.1541 (3)	0.2438 (2)	0.0200 (6)	
N3	0.3234 (4)	0.0093 (3)	0.4248 (2)	0.0170 (6)	
C1	−0.0559 (5)	0.5013 (4)	0.2716 (3)	0.0196 (7)	
H1A	−0.1291	0.5928	0.2934	0.029*	
H1B	−0.0926	0.4099	0.3329	0.029*	
H1C	−0.0808	0.5014	0.1948	0.029*	
C2	0.3814 (5)	0.5474 (4)	0.3718 (3)	0.0206 (7)	
H2A	0.3203	0.6445	0.3888	0.031*	
H2B	0.5127	0.5542	0.3327	0.031*	
H2C	0.3791	0.4651	0.4465	0.031*	
C3	0.4119 (5)	0.0140 (4)	0.3117 (3)	0.0203 (7)	
H3A	0.4696	−0.0738	0.2852	0.024*	
C4	0.2588 (5)	0.1574 (3)	0.4269 (2)	0.0158 (7)	
C5	0.2958 (5)	−0.1263 (3)	0.5236 (2)	0.0220 (8)	
H5A	0.2864	−0.1025	0.5989	0.033*	
H5B	0.4033	−0.2079	0.5155	0.033*	
H5C	0.1793	−0.1599	0.5231	0.033*	
C6	0.7959 (5)	0.2468 (4)	0.1196 (3)	0.0234 (8)	
H6A	0.7568	0.2827	0.1912	0.035*	
H6B	0.9060	0.2915	0.0701	0.035*	
H6C	0.8288	0.1351	0.1427	0.035*	
C7	0.3661 (5)	0.2266 (4)	−0.0232 (3)	0.0218 (7)	
H7A	0.2444	0.2463	0.0280	0.033*	
H7B	0.4044	0.1162	−0.0117	0.033*	
H7C	0.3538	0.2769	−0.1068	0.033*	
C8	0.1025 (5)	0.8628 (3)	0.1446 (3)	0.0236 (8)	
H8A	0.0946	0.8810	0.2218	0.035*	
H8B	−0.0223	0.8510	0.1364	0.035*	
H8C	0.1450	0.9496	0.0804	0.035*	

### Atomic displacement parameters (Å^2^)

**Table:**

	*U*^11^	*U*^22^	*U*^33^	*U*^12^	*U*^13^	*U*^23^
Sn1	0.01333 (13)	0.01317 (12)	0.01025 (11)	−0.00160 (9)	−0.00007 (8)	−0.00313 (8)
Sn2	0.01480 (13)	0.01053 (11)	0.01182 (11)	−0.00096 (9)	0.00021 (8)	−0.00231 (8)
S1	0.0276 (5)	0.0250 (4)	0.0145 (4)	−0.0043 (4)	0.0027 (3)	−0.0063 (3)
O1	0.0232 (14)	0.0111 (10)	0.0144 (10)	0.0027 (10)	0.0031 (9)	−0.0012 (8)
O2	0.0194 (13)	0.0125 (10)	0.0090 (9)	−0.0008 (9)	0.0043 (9)	−0.0016 (8)
N1	0.0175 (16)	0.0139 (13)	0.0134 (12)	−0.0016 (11)	−0.0005 (11)	−0.0031 (10)
N2	0.0265 (18)	0.0168 (14)	0.0144 (12)	−0.0033 (13)	0.0013 (12)	−0.0047 (11)
N3	0.0187 (16)	0.0154 (13)	0.0168 (12)	−0.0066 (12)	−0.0015 (11)	−0.0029 (10)
C1	0.0143 (19)	0.0224 (17)	0.0223 (16)	−0.0030 (14)	−0.0044 (14)	−0.0055 (13)
C2	0.024 (2)	0.0227 (17)	0.0196 (16)	−0.0058 (15)	−0.0079 (14)	−0.0071 (13)
C3	0.027 (2)	0.0149 (16)	0.0173 (15)	−0.0011 (15)	0.0006 (14)	−0.0065 (13)
C4	0.0167 (19)	0.0150 (15)	0.0151 (15)	−0.0033 (13)	−0.0060 (13)	−0.0004 (12)
C5	0.030 (2)	0.0157 (16)	0.0172 (15)	−0.0087 (15)	−0.0033 (14)	0.0029 (13)
C6	0.023 (2)	0.0249 (18)	0.0221 (17)	−0.0021 (15)	−0.0051 (15)	−0.0062 (14)
C7	0.018 (2)	0.0245 (17)	0.0253 (17)	−0.0083 (15)	−0.0032 (14)	−0.0073 (14)
C8	0.028 (2)	0.0155 (16)	0.0206 (16)	0.0016 (15)	0.0046 (14)	−0.0057 (13)

### Geometric parameters (Å, °)

**Table:**

Sn1—O2	2.0235 (19)	N3—C5	1.453 (4)
Sn1—C2	2.110 (3)	C1—H1A	0.9800
Sn1—C1	2.114 (3)	C1—H1B	0.9800
Sn1—O1	2.1637 (19)	C1—H1C	0.9800
Sn1—N1	2.239 (2)	C2—H2A	0.9800
Sn2—O2^i^	2.0717 (19)	C2—H2B	0.9800
Sn2—O2	2.1014 (19)	C2—H2C	0.9800
Sn2—C7	2.110 (3)	C3—H3A	0.9500
Sn2—C6	2.110 (3)	C5—H5A	0.9800
Sn2—O1^i^	2.202 (2)	C5—H5B	0.9800
Sn2—N2	2.757 (3)	C5—H5C	0.9800
S1—C4	1.702 (3)	C6—H6A	0.9800
O1—C8	1.410 (4)	C6—H6B	0.9800
O1—Sn2^i^	2.202 (2)	C6—H6C	0.9800
O2—Sn2^i^	2.0717 (19)	C7—H7A	0.9800
N1—C4	1.338 (4)	C7—H7B	0.9800
N1—N2	1.392 (3)	C7—H7C	0.9800
N2—C3	1.304 (4)	C8—H8A	0.9800
N3—C3	1.356 (4)	C8—H8B	0.9800
N3—C4	1.366 (4)	C8—H8C	0.9800
			
O2—Sn1—C2	113.32 (11)	Sn1—C1—H1B	109.5
O2—Sn1—C1	115.63 (10)	H1A—C1—H1B	109.5
C2—Sn1—C1	130.58 (13)	Sn1—C1—H1C	109.5
O2—Sn1—O1	73.45 (8)	H1A—C1—H1C	109.5
C2—Sn1—O1	92.65 (10)	H1B—C1—H1C	109.5
C1—Sn1—O1	94.71 (11)	Sn1—C2—H2A	109.5
O2—Sn1—N1	83.59 (8)	Sn1—C2—H2B	109.5
C2—Sn1—N1	96.97 (11)	H2A—C2—H2B	109.5
C1—Sn1—N1	94.75 (11)	Sn1—C2—H2C	109.5
O1—Sn1—N1	157.03 (8)	H2A—C2—H2C	109.5
O2^i^—Sn2—O2	74.62 (8)	H2B—C2—H2C	109.5
O2^i^—Sn2—C7	106.60 (11)	N2—C3—N3	111.7 (3)
O2—Sn2—C7	100.71 (11)	N2—C3—H3A	124.2
O2^i^—Sn2—C6	109.11 (11)	N3—C3—H3A	124.2
O2—Sn2—C6	99.99 (10)	N1—C4—N3	107.2 (2)
C7—Sn2—C6	142.30 (13)	N1—C4—S1	126.7 (2)
O2^i^—Sn2—O1^i^	71.73 (7)	N3—C4—S1	126.0 (2)
O2—Sn2—O1^i^	146.35 (8)	N3—C5—H5A	109.5
C7—Sn2—O1^i^	89.12 (11)	N3—C5—H5B	109.5
C6—Sn2—O1^i^	90.78 (11)	H5A—C5—H5B	109.5
O2^i^—Sn2—N2	148.29 (8)	N3—C5—H5C	109.5
O2—Sn2—N2	73.68 (7)	H5A—C5—H5C	109.5
C7—Sn2—N2	78.86 (10)	H5B—C5—H5C	109.5
C6—Sn2—N2	77.22 (11)	Sn2—C6—H6A	109.5
O1^i^—Sn2—N2	139.97 (7)	Sn2—C6—H6B	109.5
C8—O1—Sn1	128.80 (18)	H6A—C6—H6B	109.5
C8—O1—Sn2^i^	126.87 (17)	Sn2—C6—H6C	109.5
Sn1—O1—Sn2^i^	102.34 (8)	H6A—C6—H6C	109.5
Sn1—O2—Sn2^i^	112.30 (9)	H6B—C6—H6C	109.5
Sn1—O2—Sn2	142.18 (10)	Sn2—C7—H7A	109.5
Sn2^i^—O2—Sn2	105.38 (8)	Sn2—C7—H7B	109.5
C4—N1—N2	109.3 (2)	H7A—C7—H7B	109.5
C4—N1—Sn1	125.19 (19)	Sn2—C7—H7C	109.5
N2—N1—Sn1	125.43 (17)	H7A—C7—H7C	109.5
C3—N2—N1	105.4 (2)	H7B—C7—H7C	109.5
C3—N2—Sn2	139.7 (2)	O1—C8—H8A	109.5
N1—N2—Sn2	114.07 (17)	O1—C8—H8B	109.5
C3—N3—C4	106.5 (2)	H8A—C8—H8B	109.5
C3—N3—C5	127.0 (3)	O1—C8—H8C	109.5
C4—N3—C5	126.5 (3)	H8A—C8—H8C	109.5
Sn1—C1—H1A	109.5	H8B—C8—H8C	109.5
			
O2—Sn1—O1—C8	161.3 (3)	O2—Sn1—N1—N2	−7.7 (2)
C2—Sn1—O1—C8	−85.1 (3)	C2—Sn1—N1—N2	−120.5 (3)
C1—Sn1—O1—C8	46.0 (3)	C1—Sn1—N1—N2	107.6 (3)
N1—Sn1—O1—C8	160.0 (3)	O1—Sn1—N1—N2	−6.4 (4)
O2—Sn1—O1—Sn2^i^	−3.20 (8)	C4—N1—N2—C3	−0.1 (4)
C2—Sn1—O1—Sn2^i^	110.35 (12)	Sn1—N1—N2—C3	−176.4 (2)
C1—Sn1—O1—Sn2^i^	−118.56 (11)	C4—N1—N2—Sn2	−171.7 (2)
N1—Sn1—O1—Sn2^i^	−4.5 (3)	Sn1—N1—N2—Sn2	11.9 (3)
C2—Sn1—O2—Sn2^i^	−82.07 (13)	O2^i^—Sn2—N2—C3	−177.7 (3)
C1—Sn1—O2—Sn2^i^	90.87 (13)	O2—Sn2—N2—C3	−176.5 (4)
O1—Sn1—O2—Sn2^i^	3.60 (9)	C7—Sn2—N2—C3	78.7 (4)
N1—Sn1—O2—Sn2^i^	−176.92 (12)	C6—Sn2—N2—C3	−71.9 (4)
C2—Sn1—O2—Sn2	92.7 (2)	O1^i^—Sn2—N2—C3	3.7 (4)
C1—Sn1—O2—Sn2	−94.4 (2)	O2^i^—Sn2—N2—N1	−10.1 (3)
O1—Sn1—O2—Sn2	178.3 (2)	O2—Sn2—N2—N1	−8.9 (2)
N1—Sn1—O2—Sn2	−2.17 (19)	C7—Sn2—N2—N1	−113.7 (2)
O2^i^—Sn2—O2—Sn1	−175.0 (3)	C6—Sn2—N2—N1	95.6 (2)
C7—Sn2—O2—Sn1	80.6 (2)	O1^i^—Sn2—N2—N1	171.27 (18)
C6—Sn2—O2—Sn1	−67.7 (2)	N1—N2—C3—N3	−0.2 (4)
O1^i^—Sn2—O2—Sn1	−174.57 (14)	Sn2—N2—C3—N3	168.0 (2)
N2—Sn2—O2—Sn1	5.68 (17)	C4—N3—C3—N2	0.4 (4)
O2^i^—Sn2—O2—Sn2^i^	0.0	C5—N3—C3—N2	178.3 (3)
C7—Sn2—O2—Sn2^i^	−104.44 (12)	N2—N1—C4—N3	0.3 (4)
C6—Sn2—O2—Sn2^i^	107.23 (12)	Sn1—N1—C4—N3	176.7 (2)
O1^i^—Sn2—O2—Sn2^i^	0.4 (2)	N2—N1—C4—S1	178.7 (2)
N2—Sn2—O2—Sn2^i^	−179.36 (12)	Sn1—N1—C4—S1	−5.0 (4)
O2—Sn1—N1—C4	176.5 (3)	C3—N3—C4—N1	−0.5 (4)
C2—Sn1—N1—C4	63.7 (3)	C5—N3—C4—N1	−178.4 (3)
C1—Sn1—N1—C4	−68.2 (3)	C3—N3—C4—S1	−178.8 (3)
O1—Sn1—N1—C4	177.8 (2)	C5—N3—C4—S1	3.3 (5)

Symmetry codes: (i) −*x*+1, −*y*+1, −*z*.

## References

[bb1] Agilent Technologies (2010). *CrysAlis PRO* Agilent Technologies, Yarnton, England.

[bb2] Barbour, L. J. (2001). *J. Supramol. Chem.* **1**, 189–191.

[bb3] Ma, C.-L., Sun, J.-S., Zhang, R.-F. & Wang, D.-Q. (2007). *J. Organomet. Chem.* **692**, 4029–4042.

[bb4] Sheldrick, G. M. (2008). *Acta Cryst.* A**64**, 112–122. 10.1107/S0108767307043930 18156677

[bb5] Westrip, S. P. (2010). *J. Appl. Cryst.* **43**, 920–925.

[bb6] Yu, H.-X., Ma, J.-F., Xu, G.-H., Li, S.-L., Yang, J., Liu, Y.-Y. & Chen, Y.-X. (2006). *J. Organomet. Chem.* **691**, 3531–3539.

